# Exploring bone formation mechanism and pattern during RANKL inhibition in a fibrous dysplasia mouse model

**DOI:** 10.1038/s42003-026-10097-z

**Published:** 2026-04-22

**Authors:** Giorgia Farinacci, Ilenia Coletta, Biagio Palmisano, Emanuela Spica, Chiara Tavanti, Samuele Di Cristofano, Samantha Donsante, Marta Serafini, Tiziana Borsello, Mary Anna Venneri, Alessandro Corsi, Debora Salerno, Benedetta Donati, Alessia Ciarrocchi, Domenico Raimondo, Mara Riminucci

**Affiliations:** 1 Department of Experimental Medicine, Sapienza University of Rome, Rome, Italy; 2 IRCCS Neuromed, Pozzilli (IS), Italy; 3 Department of Molecular Medicine, Sapienza University of Rome, Rome, Italy; 4 Department of Molecular Medicine, Sapienza University of Rome, Laboratory Affiliated to Istituto Pasteur-Fondazione Cenci Bolognetti, Rome, Italy; 5 Tettamanti Center, Fondazione IRCCS San Gerardo dei Tintori, Monza, Italy; 6 School of Medicine and Surgery, University of Milano Bicocca, Milan, Italy; 7 Department of Pharmacological and Biomolecular Sciences, University of Milan, Milan, Italy; 8 Department of Neuroscience, Mario Negri Institute of Pharmacological Research IRCCS, Milan, Italy; 9 Laboratory of Translational Research, Azienda USL-IRCCS di Reggio Emilia, Reggio Emilia, Italy

**Keywords:** Skeleton, Diseases

## Abstract

Fibrous dysplasia (FD) of bone is a fibro-osseous disorder caused by GNAS mutations with defective osteogenic differentiation and increased bone remodeling activity. Inhibition of RANKL leads to the replacement of FD lesions with bone. However, the mechanism and pattern of deposition of the newly formed bone remain unclear. Here, we perform morphological and molecular analyses on EF1α-Gsα^R201C^ (FD) mice receiving an anti-mouse RANKL antibody. We show that, although the treatment reduces the expression of osteogenic genes, osteoblastic cells continue to produce bone matrix within FD lesions. However, bone formation does not occur in a diffuse or stochastic manner but follows an ordered spatial pattern that is restricted to the surfaces of the lesional bone. These results suggest that during RANKL inhibition the amount of intra-lesional bone trabecular surfaces is critical to the process of cell differentiation and to the skeletal improvement that FD patients may achieve during the treatment.

**Figure:**
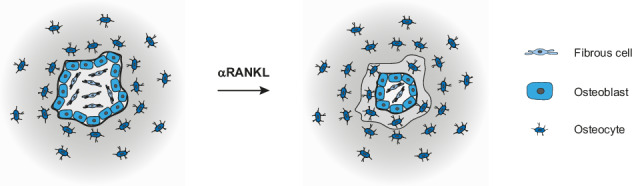

## Abstract (web summary)

Molecular analysis and calcein labeling shed light on the mechanism and pattern of intra-lesional bone formation induced by RANKL inhibition in a mouse model of fibrous dysplasia, providing information for treatment of the human disease

## Introduction

Fibrous dysplasia of bone (FD, OMIM 174800) is a genetic skeletal disease due to mutations at *GNAS*, which encodes the α subunit of the stimulatory G protein (Gsα)^1^. Most frequently, *GNAS* mutations cause the substitution of arginine 201 in the Gsα protein (UNIPROT ID: P63092) with a cysteine (R201C) or a histidine (R201H), thereby reducing its GTPase function and leading to persistent activity of the cAMP signaling pathway. FD lesions result from the disrupted homeostasis of Gsα-mutated osteoprogenitor cells in the post-natal bone marrow, which translates into the growth of a fibrotic stroma that produces hypo-mineralized bone while recruiting osteoclast precursors^2^. The pathological fibro-osseous tissue replaces normal bone and marrow at one or multiple sites, according to the somatic mosaicism caused by the post-zygotic occurrence of the mutation, and results in mechanically incompetent skeletal segments that deform and fracture and often cause untreatable pain^3–5^.

Although the deposition of pathological bone is the defining and best-known feature of FD^6^, bone resorption typically outpaces bone formation within individual lesions, making FD a disease with locally unbalanced bone remodeling. Thus, a rational and widely used pharmacological treatment consists of the administration of anti-resorptive drugs. Among these, bisphosphonates have been shown to provide some relief from FD pain^3^. However, neither in FD patients nor in mouse models, significant effect has been observed on the histopathology and progression of the disease^7,8^. Conversely, both preclinical and clinical studies have shown that inhibition of Receptor activator of nuclear factor kappa-Β ligand (RANKL), one of the most powerful osteoclastogenic cytokines^9,10^ abundantly expressed by the FD tissue^11–13^, reduces the bone turnover rate and the growth of lesions and, in some patients, also improves bone pain^8,13–17^. Interestingly, despite the downregulation of bone remodeling, intra-lesional bone formation is not suppressed by RANKL inhibition. Indeed, in both transgenic mice^8,13,14,17^ and FD patients^16,17^, lesions are variably refilled with hyper-mineralized bone, suggesting that the Gsα-mutated stromal cells complete their differentiation into functional osteoblasts. However, the fine mechanisms leading to the replacement of the fibrous tissue and the pattern of new bone formation remain unclear.

In this study, we analyzed FD lesions of EF1α-Gsα^R201C^ (FD) mice treated with different doses of an anti-mouse RANKL antibody (αRANKL). Our results demonstrate that terminal osteogenic differentiation of lesional progenitor cells during RANKL inhibition does not occur in a diffuse or stochastic manner and rather follows an ordered spatial pattern. Indeed, as shown by calcein labeling and von Kossa stain, de novo bone matrix deposition proceeds from the surface of pre-existing intra-lesional bone trabeculae to progressively replace the fibrous tissue. Translated into a clinical scenario, this pattern suggests that in FD patients, the density of bone trabeculae within each lesion may be an important determinant of the amount of bone therein produced during the treatment. Furthermore, our study shows that RANKL inhibition in mice significantly upregulates the expression of *Mmp2* in undifferentiated FD osteogenic cells, both in vivo and in vitro. Based on the involvement of this MMP in bone matrix mineralization, our results suggest a potential molecular mechanism linking RANKL to bone matrix homeostasis.

## Results

### Intra-lesion bone deposition follows osteoclast reduction in RANKL-inhibited FD mice

**Fig. 1:**
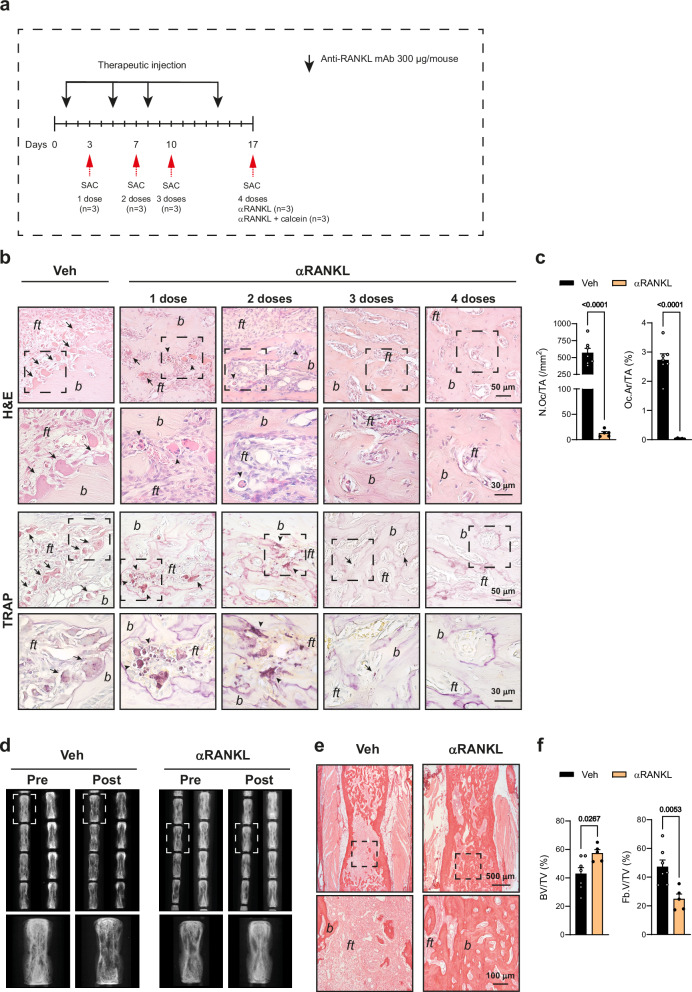
Morphological effects of RANKL inhibition on tail vertebrae of EF1α-Gsα^R201C^ mice. **a** Anti-RANKL antibody (αRANKL) treatment schedule. All control EF1α-Gsα^R201C^ mice received four injections of Vehicle (Veh). **b** Representative Haematoxylin-Eosin (H&E) and tartrate-resistant acid phosphatase (TRAP) histochemistry on tail vertebrae from EF1α-Gsα^R201C^ mice receiving Veh or different doses of αRANKL. Intra-lesion osteoclasts (arrows) become apoptotic (arrowheads) after one dose of αRANKL and progressively reduce after further doses. **c** Quantification of osteoclast parameters showing a significant reduction after four αRANKL doses compared to Veh-treated EF1α-Gsα^R201C^ mice. N.Oc/TA: number of osteoclasts per tissue area; Oc.Ar/TA: osteoclast area per tissue area. Veh *n* = 7 mice; αRANKL *n* = 5 mice. Data are represented as the mean ± SEM. Statistical analysis was performed using Student *t*-test. The exact *P*-value is reported on column bars; significance at *P* < 0.05. *b* = bone; *ft* = fibrous tissue. **d**–**f** Replacement of FD tissue with bone in the tail vertebrae of mice receiving four αRANKL doses. Radiographic analysis demonstrates the increased density of affected vertebrae at the end (Post) compared to the beginning (Pre) of the treatment (**d**). Sirius red**-**stained tissue sections (**e**) and histomorphometric analysis (**f**) confirm the higher amount of bone and lower amount of fibrous tissue in these samples compared to those from Veh-treated EF1α-Gsα^R201C^ mice. BV/TV: bone volume per tissue volume; Fb.V/TV: fibrous tissue volume per tissue volume. Veh *n* = 7 mice; αRANKL *n* = 5 mice. Data are represented as the mean ± SEM. Statistical analysis was performed using Student *t*-test. The exact *P*-value is reported on column bars; significance at *P* < 0.05. *b* = bone; *ft* = fibrous tissue.

Three-month-old FD mice with radiographic evidence of skeletal lesions were sacrificed after receiving different doses of αRANKL (Fig. 1a). Tail vertebrae were harvested and analyzed in order to assess the morphological effects of RANKL inhibition on the FD tissue and to identify lesions with ongoing fibrous to bone replacement. The earliest tissue changes induced by αRANKL treatment consisted in the appearance of apoptotic bodies in both mono and multi-nucleated TRAP positive cells, as observed in the vertebrae of mice that received either one or two doses (Fig. 1b). The number of multinucleated osteoclasts started to decline after the third αRANKL dose and was significantly reduced compared to the vehicle (Veh)-group in FD mice receiving the four-dose treatment (Fig. 1b, c) when the process of new bone formation became evident. Indeed, comparison of radiographic images of individual mice taken at the beginning and at the end of αRANKL administration showed a reduction in the number and size of lytic areas (Fig. 1d), whereas histomorphometry revealed significant differences in the amount of bone (increased) and fibrous tissue (decreased) in lesions of αRANKL- compared to Veh-treated FD mice (Fig. 1e, f). Based on these findings, tail vertebrae from the four-dose experimental group were used for subsequent NanoString gene expression profiling.

### RANKL inhibition downregulates osteogenic genes and increases *Mmp2* in mouse FD lesions

Following the identification of αRANKL-treated vertebrae with ongoing refilling of lytic areas, we analyzed the corresponding samples to evaluate treatment-induced changes in gene expression. To this aim, total mRNA was extracted from formalin-fixed-decalcified-paraffin-embedded (FFDPE) tail vertebrae of αRANKL- and Veh-treated FD mice and untreated wild-type (WT) littermates and analyzed using NanoString nCounter technology with a multiplex Custom CodeSet. The selected genes were relevant to osteoclastogenesis, matrix remodeling, osteoblast-osteoclast crosstalk and osteogenesis.

**Fig. 2:**
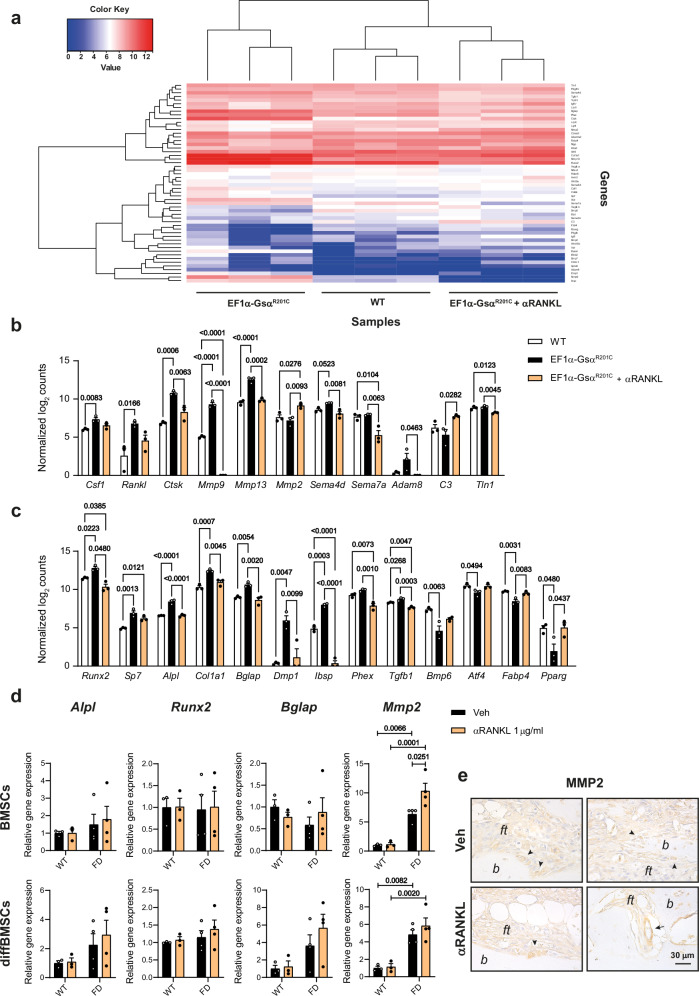
Molecular changes induced in vivo and in vitro by RANKL inhibition. **a** Heatmap representation of gene expression data obtained from FFDPE tail vertebrae. The relative abundance of transcripts is indicated by the colour (blue, lower; red, higher). **b**, **c** Column bars representation of gene expression analysis of genes related to osteoclasts, matrix remodeling, osteoblast-osteoclast crosstalk (**b**) and genes involved in osteogenic and adipogenic differentiation (**c**) in WT mice and EF1α-Gsα^R201C^ mice treated with four doses of either Veh or αRANKL. WT *n* = 3 mice; EF1α-Gsα^R201C^ + Veh *n* = 3 mice; EF1α-Gsα^R201C^ + αRANKL *n* = 3 mice. Data are represented as the mean ± SEM. Statistical analysis was performed using One-way ANOVA followed by a Tukey’s multiple comparison test. The exact *P*-value is reported on column bars; significance at *P* < 0.05. **d** qPCR performed on undifferentiated (BMSCs) and differentiated (diffBMSCs) stromal cells isolated from tail vertebrae of WT and EF1α-Gsα^R201C^ (FD) mice and treated with Veh or 1 µg/ml αRANKL. WT + Veh *n* = 3 mice, WT + αRANKL 1 µg/ml *n* = 3 mice; FD + Veh *n* = 4 mice, FD + αRANKL 1 µg/ml *n* = 4 mice. Data are represented as the mean ± SEM. Statistical analysis was performed using Two-way ANOVA followed by a Tukey’s multiple comparison test. The exact *P*-value is reported on column bars; significance at *P* < 0.05. **e** Immunolocalization of MMP2 in EF1α-Gsα^R201C^ mice treated with Veh or αRANKL. *b* = bone; *ft* = fibrous tissue.

Unsupervised clustering (Fig. 2a) and silhouette plot analyses (Supplementary Fig. 1a) revealed two distinct clusters, one corresponding to Veh-treated FD mice and the other including αRANKL-treated FD and WT mice. Compared to WT, Veh-treated FD mice exhibited increased expression of several bone remodeling-related genes, including *Csf1*, *Rankl*, *Ctsk* and *Sema4d*, while other transcripts such as *Sema7a*, *C3* and *Tln1* remained unchanged (Fig. 2b). Furthermore, *Mmp9* and *Mmp13* mRNAs were also upregulated in Veh-treated FD samples compared to WT, whereas *Mmp2* mRNA remained unchanged despite lesion development. Osteogenic differentiation markers were also elevated in Veh-treated FD mice, with the exception of *Bmp6* and *Atf4*, which were decreased compared to WT (Fig. 2c).

Expression of the two adipogenic markers, *Pparg* and *Fabp4*, was markedly lower than WT, consistent with the absence of marrow adipocytes within the FD tissue (Fig. 2c).

Following αRANKL treatment, osteoclast-derived gene transcripts became low or undetectable, although *Csf1* and *Rankl* expression was not affected (Fig. 2b). Among matrix remodeling genes, *Mmp9* expression was nearly abolished and *Mmp13* returned to the WT levels. Interestingly, *Mmp2*, a metalloproteinase implicated in bone mineralization and density^18,19^, was significantly upregulated in αRANKL-treated FD vertebrae compared with both Veh-treated FD and WT mice (Fig. 2b). Despite histological evidence of intra-lesion bone deposition, osteogenic genes expression was lower in αRANKL- compared to Veh-treated FD mice, and in some cases even lower than WT samples. Of note, this reduction involved both early (*Runx2* and *Alpl*) and late (*Dmp1* and *Phex*) osteogenic transcripts (Fig. 2c). Furthermore, in parallel with the decrease in the amounts of osteogenic transcripts, the αRANKL treatment restored the levels of mRNAs for *Pparg* and *Fabp4* (Fig. 2c), in agreement with the focal evidence of developing multilocular adipocytes in this group of vertebrae (Supplementary Fig. 2). NanoString results were validated by qPCR analysis of selected genes using fresh vertebral tissue from 3-month-old FD and WT mice (Supplementary Fig. 1b).

### αRANKL stimulates *Mmp2* expression in mouse FD osteoprogenitor cells

To assess whether αRANKL directly modulated gene expression in FD cells, we performed in vitro studies. Cells isolated from the tail vertebrae of Veh-treated FD mice and WT littermates were incubated in either standard growth medium or osteogenic medium for 14 days and treated with 1 µg/ml of αRANKL for 6 h. qPCR analysis was then carried out to assess the level of expression of representative osteogenic genes, including *Runx2* and *Alpl* for early osteogenic commitment and *Bglap* for late stages of osteogenic differentiation, as well as *Mmp2*. Furthermore, we analyzed the expression of *Rankl* and *Rank* transcripts. Across all conditions, αRANKL treatment had no significant impact on the expression of the osteogenic genes (Fig. 2d), although we observed a slight increase in the level of the osteocalcin transcript in treated compared to untreated FD cell cultures. Importantly, αRANKL induced a significant increase in *Mmp2* mRNA in undifferentiated FD stromal cells (Fig. 2d). As previously reported, both *Rankl*^12,13^ and *Rank*^20^ transcripts were detected in cell cultures although at a very low levels compared to *Alpl* (Supplementary Fig. 3).

In agreement with in vitro findings, immunolocalization of MMP2 showed a positive signal in some undifferentiated perivascular stromal cells only in bone lesions of αRANKL-treated FD mice, whereas MMP2 expression in osteoblasts was overall comparable between αRANKL- and Veh-treated FD mice (Fig. 2e).

### Bone formation in mouse FD lesions during RANKL inhibition is restricted to bone surfaces

**Fig. 3:**
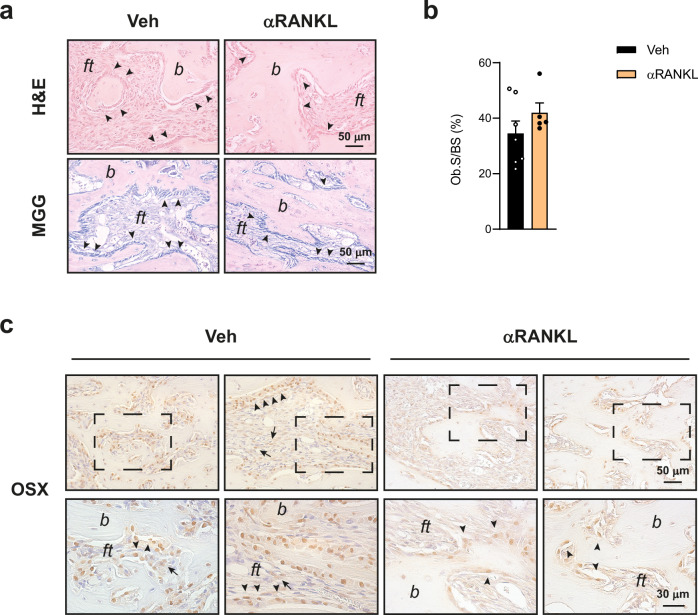
Osteoblast distribution in tail vertebrae of Veh- and αRANKL-treated EF1α-Gsα^R201C^ mice. **a** Haematoxylin-Eosin (H&E) and May Grünwald-Giemsa (MGG) stains showing osteoblasts (arrowheads) within tail lesions of Veh- and αRANKL-treated EF1α-Gsα^R201C^ mice. *b* = bone; *ft* = fibrous tissue. **b** Histomorphometric analysis reveals comparable values of osteoblast surface per bone surface (Ob.S/BS) in the tail vertebrae of αRANKL- compared to Veh-treated EF1α-Gsα^R201C^ mice. Veh *n* = 7 mice; αRANKL *n* = 5 mice. Data are represented as the mean ± SEM. Statistical analysis was performed using Student *t*-test. **c** Immunolocalization of OSX in EF1α-Gsα^R201C^ mice treated with Veh or αRANKL showing the expression of OSX in osteoblasts (arrowheads) bordering the bone surface and in a few cells within fibrous tissue (arrows). *b* = bone; *ft* = fibrous tissue.

**Fig. 4:**
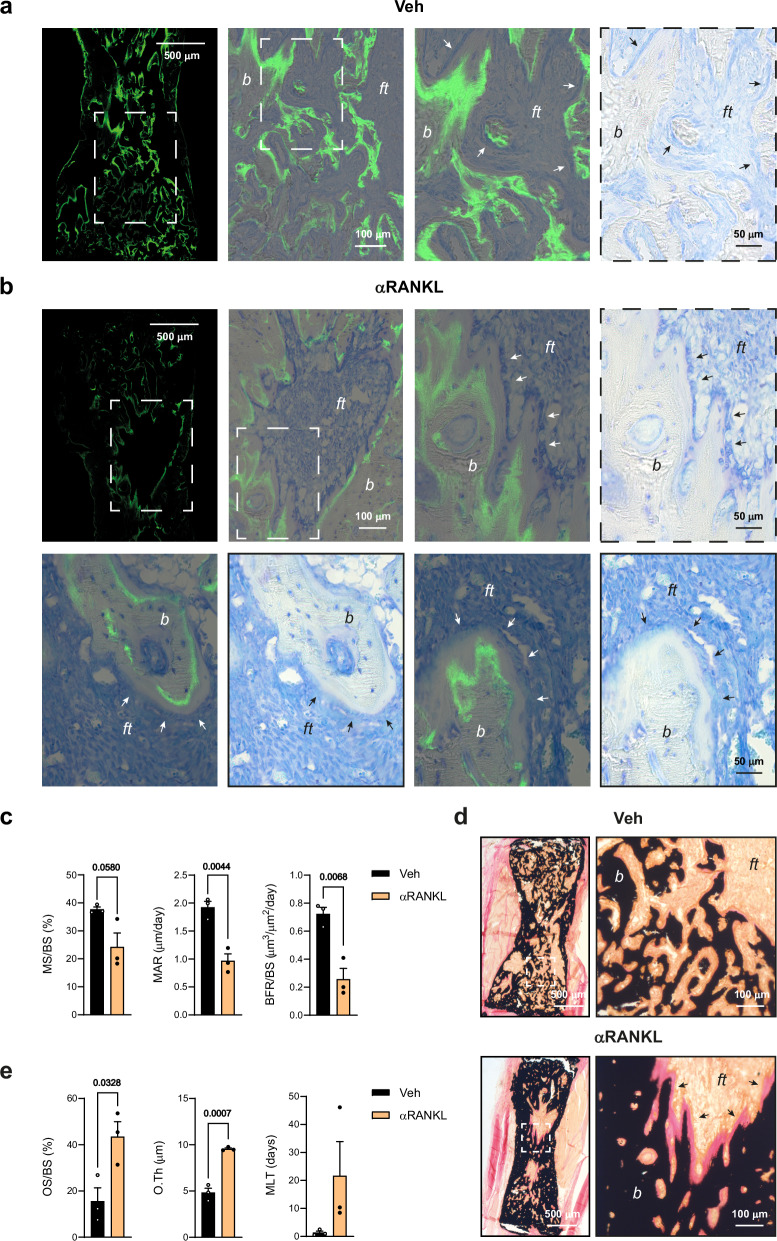
Pattern of bone formation in affected tail vertebrae of Veh- and αRANKL-treated EF1α-Gsα^R201C^ mice. **a**, **b** Tail vertebrae of EF1α-Gsα^R201C^ mice labeled with calcein and stained with Toluidine Blue. Fluorescence microscopy reveals a smeared pattern of fluorescence in Veh-treated EF1α-Gsα^R201C^ mice (**a**). After αRANKL treatment, calcein labeling is detected as a single line, with focally smeared areas, on all intra-lesional bone surfaces where newly formed bone bordered by osteoblasts (arrows) is also observed (**b**). Note the absence of fluorescent labeling within the fibrous tissue. Veh *n* = 3 mice; αRANKL *n* = 3 mice. *b* = bone; *ft* = fibrous tissue. **c** Bone dynamic histomorphometric analysis of mineralizing surface per bone surface (MS/BS), mineral apposition rate (MAR) and bone formation rate (normalized for BS, BFR/BS) performed on undecalcified samples. Veh *n* = 3 mice; αRANKL *n* = 3 mice. Data are represented as the mean ± SEM. Statistical analysis was performed using Student *t*-test. The exact *P*-value is reported on column bars; significance at *P* < 0.05. **d** Von Kossa/van Gieson-stained sections of undecalcified tail vertebrae showing the presence of osteoid seams (arrows) on the bone surface of αRANKL-treated EF1α-Gsα^R201C^ mice. *b* = bone; *ft* = fibrous tissue. **e** Histomorphometric analysis of osteoid surface per bone surface (OS/BS), osteoid thickness (O.Th) and mineralization lag time (MLT) performed on von Kossa**/**van Gieson-stained sections. Veh *n* = 3 mice; αRANKL *n* = 3 mice. Data are represented as the mean ± SEM. Statistical analysis was performed using Student *t*-test. The exact *P*-value is reported on column bars; significance at *P* < 0.05.

**Fig. 5:**
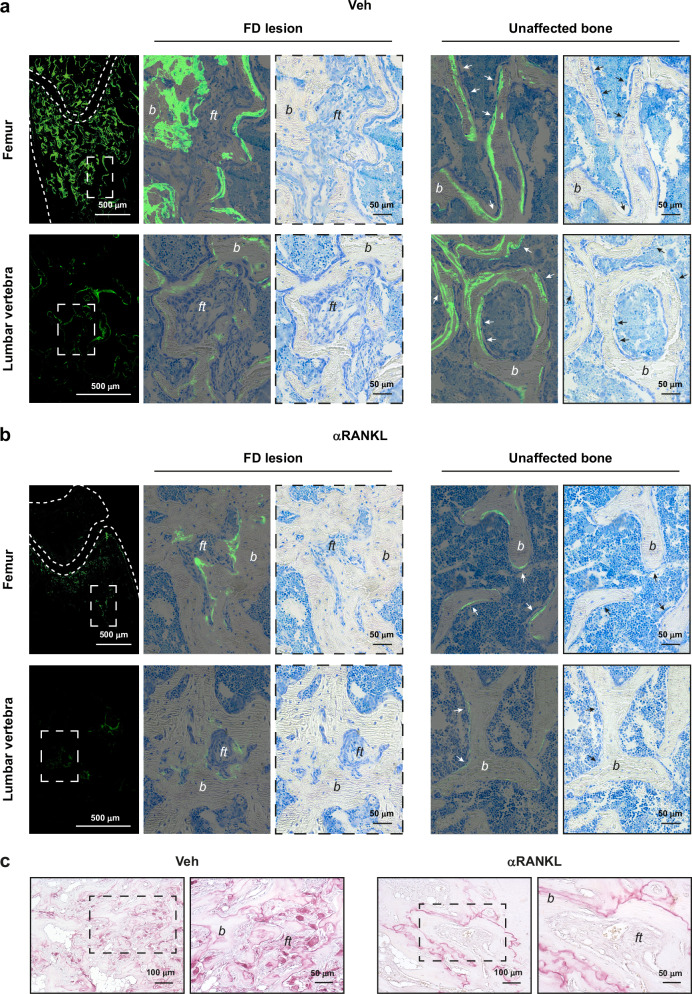
Pattern of bone formation in femurs and lumbar vertebrae of Veh- and αRANKL-treated EF1α-Gsα^R201C^ mice. **a**, **b** Femurs and lumbar vertebrae of EF1α-Gsα^R201C^ mice labeled with calcein and stained with Toluidine Blue. Numerous bright double fluorescent lines are observed on unaffected bone trabeculae of Veh-treated EF1α-Gsα^R201C^ mice (**a**, arrows). Calcein labeling is markedly reduced in αRANKL-treated EF1α-Gsα^R201C^ mice in which double fluorescent lines are rarely observed on unaffected bone trabeculae (**b**, arrows). Veh *n* = 3 mice; αRANKL *n* = 3 mice. *b* = bone; *ft* = fibrous tissue. **c** Tartrate-resistant acid phosphatase (TRAP) histochemistry of tail vertebrae showing the presence of multiple sites of bone remodeling in Veh- treated EF1α-Gsα^R201C^ mice and their reduction during αRANKL treatment. Note the bone produced on the TRAP positive reversal lines during RANKL inhibition encasing the residual fibrous tissue. *b* = bone; *ft* = fibrous tissue.

In vivo and in vitro molecular studies suggested that inhibition of RANKL in FD mice did not associate with a massive osteogenic differentiation of cells within the intra-lesional fibrous tissue. This conclusion was further supported by histomorphometry and immunophenotypic evaluation performed on the same FFDPE vertebrae used for gene expression analysis. Indeed, MGG stain did not show changes in the morphology and/or organization of cells within the fibrotic tissue (Fig. 3a), and osteoblasts remained restricted to bone surfaces with no histomorphometric differences compared to Veh-treated FD vertebrae (Fig. 3a, b). Furthermore, immunolocalization experiments confirmed that, as in Veh-treated FD samples, the expression of the transcription factor OSX remained localized to the osteoblastic cells bordering the bone trabeculae (arrowheads; Fig. 3c), with only a few, sparse positive cells within the fibrous tissue (arrows; Fig. 3c). These results suggested that RANKL inhibition did not lead to bone formation by affecting the osteogenic differentiation status of fibroblast-like cells in the lesional tissue. Based on these observations, calcein labeling was performed to further clarify the pattern of bone matrix deposition and mineralization. In the tail vertebrae (Fig. 4a), femurs and lumbar vertebrae (Fig. 5a) of the Veh group, the green fluorescence was bright, extensive and focally smeared on lesional bone trabeculae. This appearance was consistent with the florid deposition of bone, related to the enhanced bone remodeling, and the osteomalacic changes that typically occur in FD, both in mice^21^ and patients^22,23^. As expected, double labeling was observed on femoral bone trabeculae spared from the disease and in unaffected lumbar vertebrae (Fig. 5a). In all the examined skeletal segments, the intensity and distribution of the fluorescent labeling was overall reduced in αRANKL-treated compared to Veh-treated mice (Figs. 4b and 5b). Mineralizing surface, mineral apposition rate and bone formation rate measured at lesional sites of caudal vertebrae were markedly reduced in αRANKL-treated mice (Fig. 4c), reflecting the downregulation of bone remodeling. Accordingly, in unaffected bone trabeculae of femurs and lumbar vertebrae of αRANKL-treated FD mice, the frequency of double labeling was very low and the distance between the double label was much shorter than Veh-treated bone segments (Fig. 5b). Nonetheless, within the residual pathological tissue of αRANKL-treated mice, calcein labeling was detected on most bone trabeculae, where it occurred as single fluorescent lines with focally smeared areas (Figs. 4b and 5b). Of note, no deposit of the fluorochrome was ever observed within the fibrous tissue (Figs. 4b and 5b). In the same samples, Toluidine Blue (Fig. 4b) and von Kossa (Fig. 4d) stains revealed the presence of osteoid seams covering the entire interface between the mineralized bone and the fibrous tissue (Fig. 4d). Histomorphometric analysis revealed a significant higher osteoid surface and thickness in αRANKL-treated mice compared to Veh (Fig. 4e). These results, together with the higher mineralization lag time (Fig. 4e), indicated a delayed mineralization of the newly formed bone, suggesting that the treatment did not fully restore the mineralization capacity of osteoblasts in FD tissue.

The ordered occurrence of bone formation at the interface between bone surfaces and marrow fibrosis was further supported by the evidence within lytic lesions of a thick and continuous layer of bone matrix localized between the reversal lines, well highlighted by TRAP^24^, and the remaining fibrous core (Fig. 5c).

Overall, these results suggest that in αRANKL-treated FD mice, the new bone matrix was not produced randomly within FD lesions but was restricted to the surface of the pre-existing lesional bone trabeculae. This spatial pattern resulted from the activity of bone-forming cells already engaged in bone remodeling, which completed the ongoing remodeling cycles, likely followed by differentiation of osteoprogenitor cells at the interface between fibrous tissue and bone.

Altogether, these results not only clarified the mechanisms of intra-lesional bone mass gain induced by RANKL inhibition but also shed light on why it associated with a reduction rather than an increase in the level of osteogenic transcripts.

## Discussion

Inhibition of RANKL activity is currently the only therapeutic approach able to modify the tissue pathology of FD by inducing the replacement of fibrous tissue with bone. This effect has been observed in transgenic mouse models^8,13,14,17,25^ and FD patients^16,17^, and is induced by both antibodies^8,13,16,17^ and small molecules that inhibit RANKL activity^14^. Notably, this effect is not replicated by other anti-bone resorption drugs that target osteoclast function rather than formation, such as BPs^8^. However, few data are currently available on the mechanism of formation and pattern of deposition of the replacing bone matrix. In most clinical studies, FD patients’ response to the humanized anti-RANKL antibody denosumab was assessed through surrogates for bone biopsy, such as serum levels of bone turn-over markers (BTMs) and bone imaging^26–28^. However, serum BTMs, being evaluated on a systemic scale, reflect the effect of the drug on affected and non-affected skeletal segments rather than the osteogenic activity within individual FD lesions. Bone imaging, on the other side, captures treatment-dependent modifications in the density or tracer uptake of affected bones but does not reveal how the changes developed. Furthermore, patient monitoring usually starts at relatively long intervals from the beginning of the treatment, leaving its early effects unexplored. Similarly, in most of the available studies on FD transgenic mice, histological evaluation was performed at the end of the treatment rather than during drug administration. A single recent work reported histological and immunophenotypic evaluation of bone biopsies obtained from FD patients and bone samples from transgenic mice after 6 months and 4 weeks, respectively, of RANKL inhibition. In both cases, an increase in the number of SOST-positive osteocytes was observed, consistent with the increased amount of intra-lesional bone, whereas a reduction of *Runx2* and *Alpl* was observed in the mice^17^.

In our study, we monitored FD lesions of EF1α-Gsα^R201C^ mice after different doses of an anti-mouse RANKL antibody. We first observed that a significant replacement of fibrous tissue with bone could be detected only after apoptosis of pre-existing TRAP-positive osteoclasts that started shortly after the first dose and caused their significant reduction. Since it was previously reported that osteoclasts support the proliferation of FD osteoprogenitor cells^17^, we hypothesized that a reduction in the proliferation activity caused by their removal could be followed by an increase in osteogenic differentiation within the entire fibrous tissue. However, the downregulation of osteogenic markers, which was in agreement with the results of a previous study^17^, and the absence of calcein labeling in the fibrotic areas far from bone surfaces, could hardly be reconciled with this possibility. Indeed, von Kossa stain and calcein labeling demonstrated that during the anti-RANKL treatment, the osteogenic activity remained restricted to the bone surface, suggesting that refilling of pre-existing remodeling spaces is the first mechanism for the bone mass gain caused by the treatment. The refilling process is the main mechanism that underlies the effect of RANKL inhibition in denosumab-treated osteoporotic patients, in which osteogenic cells already engaged in bone formation continue to form extracellular matrix and complete the refilling of individual bone multicellular units (BMUs) before becoming quiescent^29,30^. FD is a high bone remodeling disease in which the continuous activation of resorption sites shortens the lifespan of pre-existing BMUs. In this context, the downregulation of bone remodeling caused by RANKL inhibition, confirmed by both the suppression of osteoclastogenic and osteogenic markers and the overall reduced calcein labeling, is expected to allow the refilling of an abnormally high number of BMUs, leading to an increase in bone mass higher than that observed in osteoporosis. In addition to the BMU refilling process, our results suggest a second mechanism leading to bone matrix apposition. Specifically, they suggest a progressive recruitment and differentiation of osteogenic cells from the adjacent osteogenic fibrous tissue to the bone surface. Different hypotheses can be made to explain this process. For example, it is reasonable to assume that, compared to basal conditions, in RANKL-inhibited FD lesions refilling of pre-existent resorption surfaces and lack of activation of new remodeling sites both increase the osteoconductive bone surface on which adjacent osteoprogenitor cells may convert into osteoblasts. This implies that after a first phase of remodeling-dependent bone formation, a second phase of bone deposition occurs that is modeling-based and explains the bone mass gain observed in long-term treatments^13,17^. In addition, it is possible that, although RANKL inhibition seems to reduce the overall levels of the mutated Gsα mRNA^17^, the residual expression of the mutation in differentiated osteogenic cells may act as a further stimulator of bone formation, as reported in a previous work^31^. Regardless of the mechanisms, these results may help to explain the occurrence of intra-lesion bone mass gain in FD patients treated with denosumab. They may also help to understand the factors influencing the rebound phenomenon at treatment discontinuation. Indeed, the severity of the rebound phenomenon, which is caused by resumption of osteoclast activity leading to lesion recurrence, BTM rebound and hypercalcemia, has been previously related to the skeletal improvement, i.e., amount of newly formed bone, achieved during denosumab treatment^27^.

However, it must be remarked that while in transgenic mice skeletal lesions are overall homogeneous, in FD patients they may show greater variability caused, for example, by non-conventional histology^32^. Therefore, direct translation of our results into clinical settings must take into account some important variables that are not necessarily reproduced in transgenic mice.

We previously showed that in the same EF1α-Gsα^R201C^ transgenic mouse model, the bone matrix produced during long-term RANKL inhibition was hyper-mineralized^13^. In this study, part of the newly formed bone matrix produced within lesions at early times during RANKL inhibition was still extensively unmineralized. This suggests that the mineralization status achieved in the previous study^13^ likely depended on the long secondary mineralization phase due to the persistent inhibition of bone resorption^29,30,33^. However, other factors could be involved. Our results suggest that MMP2, a matrix remodeling enzyme that is known to affect bone mineralization^18,19^, might be a potential candidate. Since MMP2 is normally produced by osteocytes and bone marrow stromal cells^34^, the enhanced amount of its transcript in treated mice likely reflected the increased number of osteocytes, resulting from new bone formation, and/or the increased production by FD osteoprogenitor cells. The latter possibility is supported by our in vitro experiments, although further studies are required to elucidate the underlying mechanisms.

Our work provides additional findings relevant to the biology and treatment of FD. We observed that *Rankl* and *Csf1*, which were found to be enriched in FD mice^13,17^, did not decrease during RANKL inhibition at the gene expression level. This suggests that during the administration of the anti-RANKL antibody, CSF1 continues to act on the monocyte lineage causing the accumulation of early osteoclastic cells and that, following treatment withdrawal, RANKL stimulates the fusion of these cells into functional osteoclasts. This molecular mechanism may contribute to the rebound effect and must be considered for the development of mitigating therapeutic strategies.

On the osteogenic side, it is worth to note that the expression level of the transcription factor *Atf4* was reduced in untreated FD lesions compared to WT bone and was restored by the anti-RANKL antibody. As ATF4 is required for the expression of late osteogenic markers, such as bone sialoprotein and osteocalcin^35^, its increase may contribute to the activity and function of mutated osteogenic cells during RANKL inhibition.

Finally, as expected, the adipogenic markers *Pparg* and *Fabp4* were downregulated in untreated FD lesions compared to WT bone. Following inhibition of osteoclastogenesis, their transcripts increased consistently with the focal histological evidence of developing adipocytes. The absence of adipocytes within FD lesions is commonly ascribed to the inability of Gsα-mutated skeletal progenitors to undertake the adipogenic program.

However, our previous work showed that normal human osteoprogenitor cells transduced with the *Gsα*^*R201C*^ sequence expressed higher levels of adipogenic markers compared to control cells^11,36^ and that in FD mice receiving long-term anti-RANKL-treatment, the adipose marrow was increased compared to IgG-treated mice^13^. Altogether, these data demonstrate that, although bone marrow adipocytes are never fully restored in treated FD lesions, Gsα-mutated skeletal progenitors preserve the ability to generate adipocytes and suggest that suppression of the RANKL reverse signaling and/or osteoclast activity may favor their adipogenic fate.

In conclusion, our study shows that during RANKL inhibition, new bone formation within FD lesions does not occur diffusely or stochastically within the fibrous tissue but follows an ordered pattern originating from bone surfaces, progressively replacing adjacent fibrous tissue. Clinically, these findings suggest that the amount of FD bone trabecular surfaces rather than fibrous tissue is a critical determinant of the bone mass gain that a given denosumab course may induce in FD patients and, as a consequence, of the severity of the rebound effect that may follow treatment discontinuation.

## Methods

The study was conducted in compliance with relevant Italian laws and Institutional guidelines, and all procedures were approved by the Institutional Animal Care and Use Committee (IACUC). The total number of animals used was 34. We have complied with all relevant ethical regulations for animal use.

### Mice and experimental groups

EF1α-Gsα^R201C^ transgenic mice (FD mice)^21^ were genotyped using the oligonucleotide sequences reported in Table 1. All mice were maintained in cabin-type isolators at standard environmental conditions (temperature 22–25 °C) with 12:12 h dark/light photoperiod and food and water provided *ad libitum*. Three-month-old FD mice were treated with 300 µg/dose (12 mg/Kg) of anti-mouse RANKL antibody (αRANKL, clone IK22/5, #BE0191, Bio X Cell, West Lebanon, NH, USA, *n* = 15 mice, 12 females and 3 males) administered intraperitoneally (Fig. 1a). Since our previous work showed that in EF1α-Gsα^R201C^ mice two doses/week of the anti-RANKL antibody led to rapid and effective conversion of FD lesions into bone^13^, in this study we maintained a two-dose schedule in the first week to efficiently start the process and then reduced to one dose/week to better monitor the effects of the treatment. FD mice were then sacrificed after 1 dose (*n* = 3 mice), 2 doses (*n* = 3 mice), 3 doses (*n* = 3 mice) or 4 doses (*n* = 3 mice). Histological evaluation was carried out on all samples. Paraffin-embedded bones of mice receiving 4 doses of the antibody were also used for molecular analysis and histomorphometry. An additional group of mice was treated with 4 doses of αRANKL (*n* = 3 mice) and 2 injections of 30 mg/kg calcein (Sigma-Aldrich, Saint Louis, MO, USA) in 2% sodium bicarbonate solution at 5 and 2 days before the sacrifice^37,38^, for histological and histomorphometric studies. αRANKL-treated FD mice were compared to 3-month-old FD mice receiving 4 injections of Vehicle (Veh, *n* = 7 mice, 6 females and 1 male) and, in molecular studies, also to 3-month-old wild-type littermates (WT) mice (*n* = 3 female mice). The number of mice used in the different experiments is reported in the figure legends. Sacrifice was carried out for all mice by carbon dioxide inhalation. Sex was not considered in the study design as it does not impact on the phenotype of FD mice. FD mice were randomly assigned to the different experimental groups.

**Table 1:** Sequence of primers used for genotyping and qPCR analysis

Genotyping	
Mouse strain	Sequence 5′ - 3′
EF1α-Gsα ^R201C^	F: GGATTACGCGTCCAACAGCG
	R: TCCCGGATGACCATGTTGTA
qPCR	
Gene	Sequence 5′ - 3′
*Alpl*	F: CCAGAAAGACACCTTGACTGTGG
	R: TCTTGTCCGTGTCGCTCACCAT
*Bglap*	F: GCAATAAGGTAGTGAACAGACTCC
	R: CCATAGATGCGTTTGTAGGCGG
*Csf1*	F: GCCTCCTGTTCTACAAGTGGAAG
	R: ACTGGCAGTTCCACCTGTCTGT
*Dmp1*	F: AGCTCTGAAGAGAGGACGGG
	R: GCTGTCCGTGTGGTCACTAT
*Gapdh*	F: CATCACTGCCACCCAGAAGACTG
	R: ATGCCAGTGAGCTTCCCGTTCAG
*Mmp2*	F: CAAGGATGGACTCCTGGCACAT
	R: TACTCGCCATCAGCGTTCCCAT
*Mmp9*	F: AGCACAACAGCTGACTACGAT
	R: ATGAACGGGAACACACAGGG
*Mmp13*	F: GATGACCTGTCTGAGGAAGACC
	R: GCATTTCTCGGAGCCTGTCAAC
*Rank*	F: GGACAACGGAATCAGATGTGGTC
	R: CCACAGAGATGAAGAGGAGCAG
*Rankl*	F: CGAGCGCAGATGGATCCTAA
(in vivo)	R: GCAGGAGTCAGGTAGTGTGT
*Rankl*	F: TGCAGGAGGATGAAACAAGC
(in vitro)	R: CATCCAACCATGAGCCTTCCA
*Rna18s*	F: CGATGCTCTTAGCTGAGTGT
	R: GGTCCAAGAATTTCACCTCT
*Runx2*	F: CCTGAACTCTGCACCAAGTCCT
	R: TCATCTGGCTCAGATAGGAGGG
*Sema4d*	F: AAGGAAGATGCCCCTTCGAC
	R: GATGGGTTCACTGCCCAAGA
*Sema7a*	F: TCTGCGTGTATTCGCTTGGT
	R: AGCCACCTCTGGGTGACTAT

### X-ray analysis

Radiographic analyses were performed at the beginning of the treatment and at the time of sacrifice using Faxitron MX-20 Specimen Radiography System (Faxitron X-ray Corp., Wheeling, IL, USA) set at 24 kV for 6 s, under anesthesia with a mixture of Zoletil 20 (Virbac SA, Carros, France) and Rompun (Bayer, Leverkusen, Germany).

### Histology and histochemistry

Skeletal segments were fixed with 4% formaldehyde in PBS pH 7.4 for 48 h at 4 °C and processed for either paraffin or methyl methacrylate (MMA) embedding.

For paraffin embedding, samples were decalcified with 10% EDTA and processed according to standard procedures. Three µm-thick sections were used for histological staining with Haematoxylin-Eosin (H&E), Sirius red, May Grünwald-Giemsa (MGG) and tartrate-resistant acid phosphatase (TRAP) histochemistry using Sigma Aldrich reagents (Sigma Aldrich)^37^. Paraffin-embedded tail vertebrae to be used for RNA extraction and NanoString analysis were selected based on histologic evaluation. For MMA embedding, samples were fixed with 4% formaldehyde solution for 24 h, dehydrated through increasing ethanol concentrations, infiltrated for 3 days with a plastic embedding solution containing MMA and then incubated at −20 °C in a polymerization mixture obtained by adding N, N-dimethyl-p-toluidine to the embedding solution^38^. Five-to-eight µm-thick sections were cut from each block and after removal of MMA with 2-methoxyethylacetate, stained with Toluidine Blue before calcein fluorescence evaluation or von Kossa/van Gieson to assess bone mineralization. All chemical reagents were purchased from Sigma Aldrich. Toluidine Blue-stained sections were analyzed by an optical microscope (Zeiss Axiophot, Jena, Germany) and calcein fluorescence was detected by a Leica Confocal Microscope (Wetzlar, Germany).

### Histomorphometry

Quantitative bone histomorphometry was conducted on tail vertebrae in a region of interest (ROI) between the two growth plates. The experiments were performed in a blinded fashion. Sirius red-stained sections were used to measure trabecular bone volume per tissue volume (BV/TV) and fibrous tissue volume per tissue volume (Fb.V/TV). TRAP-stained sections were used to measure osteoclast number per tissue area (N.Oc/TA) and osteoclast area per tissue area (Oc.Ar/TA). MGG-stained sections were used to measure osteoblast surface per bone surface (Ob.S /BS). Von Kossa/van Gieson-stained MMA sections of undecalcified tail vertebrae were used to measure osteoid surface per bone surface (OS/BS) and osteoid thickness (O.Th). Quantitative dynamic bone histomorphometry was performed on undecalcified tail vertebrae and calcein fluorescent labeling was used to measure mineralizing surface per bone surface (MS/BS), mineralizing surface per osteoid surface (MS/OS), mineral apposition rate (MAR) and to calculate mineralization lag time (MLT) and bone formation rate (normalized for BS, BFR/BS).

Images were acquired with an optical microscope (Zeiss Axiophot, Jena, Germany) and all histomorphometric analyses were performed using ImageJ software (NIH, Bethesda, MD, USA) and Adobe Photoshop. Results were reported using standard nomenclature and abbreviations^39^.

### Immunohistochemistry

Immunolocalization of matrix metalloproteinase 2 (MMP2) and osterix (OSX) was carried out using rabbit anti-mouse antibodies (anti-MMP2 #GTX104577, GeneTex, Irvine, CA, USA; anti-OSX #ab22552, Abcam, Cambridge, UK) applied at a dilution of 1:300 and 1:200, respectively, in PBS and incubated overnight at 4 °C. For MMP2 immunostaining, a heat-mediated antigen retrieval step with citrate buffer (pH 6) at 98 °C for 30 min was performed. After the application of the primary antibody, sections were repeatedly washed with PBS and incubated for 30 min with biotin-conjugated polyclonal swine anti-rabbit IgG (#E0353, Dako, Agilent, Santa Clara, CA, USA, 1:500 in PBS) and then exposed for 30 min to peroxidase-conjugated streptavidin (#P0397, Dako, Agilent, Santa Clara, CA, USA; 1:1000 in PBS). The peroxidase reaction was developed using DAB substrate kit (SK-4105, Vector Laboratories, Burlingame, CA, USA).

### Gene expression analysis by NanoString nCounter technology on formalin-fixed-decalcified-paraffin-embedded (FFDPE) bone samples

Five to ten tissue sections (20 µm thickness) were obtained from decalcified, paraffin-embedded tail vertebrae (two vertebrae per mouse). Total RNA was extracted using RNeasy FFPE Kit (#73504, Qiagen, Hilden, Germany) according to the manufacturers’ instructions. RNA concentration and purity were assessed by NanoDrop 2000 spectrophotometer (Thermo Fisher Scientific, Waltham, USA). Only samples meeting quality thresholds (A260/A280 ≥ 1.7 and A260/A230 ≥ 1.8) were included for downstream analysis.

Gene expression profiling was conducted using NanoString nCounter platform with a custom-designed CodeSet targeting 54 genes involved in osteoclastogenesis, matrix remodeling, osteoblast-osteoclast cross-talk and osteogenesis (Supplementary Data 1). Raw gene counts were analyzed using nSolver Analysis Software 4.0 (NanoString Technologies, Seattle, WA, USA). Samples were selected based on NanoString quality control metrics: percentage of fields of view (FOV) read (>75%), binding density (between 0.05 and 2.25), positive control linearity (>0.95) and positive control limit of detection (>2)^40,41^.

Raw counts were normalized using the geometric mean of the three housekeeping genes (*Actb*, *Cltc*, *Cnot10*) that showed an average count >150, after validation against positive and negative controls. Normalized gene counts were log_2_ transformed and used for downstream analyses.

### Clustering analysis and Silhouette plot

Gene expression data were visualized using heatmap and hierarchical clustering analysis of both samples and genes was performed with “heatmap.2” function from the *gplots* package in R (RStudio). To determine the optimal number of clusters for sample classification, silhouette plot analysis was performed using *fviz_nbclust* function from the *factoextra* and *NbClust* packages.

### Cell cultures and osteogenic differentiation

Osteogenic cells were isolated from tail vertebrae of 5-month-old WT (*n* = 3 mice) and FD (*n* = 4 mice) male mice. Briefly, after removal of skin and tendons, tail vertebrae were placed in HBSS (Gibco, Thermo Fisher Scientific) with 2 mg/ml collagenase type II (Gibco, Thermo Fisher Scientific) and incubated in a water bath for 15 min at 200 relative centrifugal force (rcf), to remove the outer layer of periosteum. Then, they were minced and further digested with 2 mg/ml collagenase type II solution in HBSS and were incubated in a water bath at 100 rcf for 1 h. After digestion, to obtain a single cell suspension, samples were filtered through a 100 µm nylon mesh, centrifuged at 1300 rcf for 5 min, resuspended in 10 ml of complete culture medium consisting of αMEM (Sigma-Aldrich) supplemented with 20% FBS (Thermo Fisher Scientific), 1% L-glutamine (L-gln, Sigma-Aldrich) and 1% penicillin/streptomycin (P/S, Sigma-Aldrich), filtered again through a 70 µm nylon mesh and centrifuged at 1300 rcf for 6 min. Cells were resuspended in complete culture medium and grown in 100 mm culture dishes. For osteogenic differentiation, cells were plated in 12-well plates at the density of 3 × 10^4^ cells/well and incubated for 14 days in medium containing complete αMEM supplemented with 4 mM β-glycerophosphate (Sigma-Aldrich) and 50 μg/ml of ascorbic acid (Sigma-Aldrich). Undifferentiated and differentiated cells were treated with either 1 μg/ml of αRANKL Ab or Veh for 6 h in FBS-free medium.

### qPCR analysis

Quantitative PCR (qPCR) was performed on both fresh tissues from tail vertebrae and cells induced to osteogenic differentiation in vitro. Tail vertebrae of 3-month-old WT (*n* = 4 mice) and FD (*n* = 4 mice) female mice were snap frozen in liquid nitrogen and homogenized by using Mikro-Dismembrator U (Gottingen, Germany). Total RNA was isolated from tissues and cells using the TRIzol Reagent protocol (Invitrogen, Thermo Fisher Scientific). Reverse transcription was performed by using Takara PrimeScript™ RT Reagent Kit with gDNA Eraser (Takara, Japan) according to the manufacturers’ instructions. qPCR analysis was conducted on cDNA samples with a 7500 Fast Real-Time PCR System (Applied Biosystem, Waltham, Massachusetts, USA), performed using PowerUP Sybr Green (Thermo Fisher Scientific) and expression levels of each gene were normalized to *Gapdh* or *Rna18s* expression. The primers used are listed in Table 1.

### Statistics and reproducibility

Data are represented as column bars showing the mean ± SEM and individual data points. Each dot represents a mouse. Unpaired two-tailed Student *t*-test was used both for histomorphometric analyses to compare Veh- and αRANKL-treated FD mice and for dynamic bone histomorphometry to compare Veh- and αRANKL-treated FD mice. One-way ANOVA followed by a Tukey’s multiple comparison test was used to compare WT and Veh- and αRANKL-treated FD mice for NanoString gene expression analysis. For gene expression analysis performed on fresh tail vertebrae to validate NanoString results, WT and FD mice were compared using unpaired two-tailed Student *t*-test. Two-way ANOVA followed by a Tukey’s multiple comparison test was used for in vitro experiments performed on undifferentiated (BMSCs) and differentiated (diffBMSCs) stromal cells isolated from tail vertebrae of WT and FD mice and treated with Veh or 1 µg/ml αRANKL. For in vitro experiments, isolated RNA from technical replicates (culture wells) was pooled to obtain cDNA for each biological replicate (mouse) that is reported in the graphs as a single dot. In all experiments a *P*-value less than 0.05 was considered statistically significant. All graphs and statistical analyses were performed using GraphPad Prism version 8 (GraphPad Software, La Jolla, CA, USA). All animals and data points were used during the experiments.

### Reporting summary

Further information on research design is available in the Nature Portfolio Reporting Summary linked to this article.

## Supplementary information

Supplementary Information

Description of Additional Supplementary Files

Supplementary Data 1

Supplementary Data 2

Supplementary Data 3

Supplementary Data 4

Supplementary Data 5

Reporting Summary

## Data Availability

All the data supporting the findings of this study, including numerical source data for all graphs present in both the main and Supplementary Figs., are provided in Supplementary Data 2–5. All the other data will be available from the corresponding author on reasonable request.
