# Supplementary material for: Exploring bone formation mechanism and pattern during RANKL inhibition in a fibrous dysplasia mouse model
Source: Commun Biol. 2026 Apr 22;9:1088. doi: 10.1038/s42003-026-10097-z (PMC13463084; doi:10.1038/s42003-026-10097-z)
Supplement: Supplementary file 1 [file 42003_2026_10097_MOESM1_ESM.pdf]

Exploring bone formation mechanism and pattern during RANKL inhibition in a fibrous dysplasia mouse model

Giorgia Farinacci [1], Ilenia Coletta [2], Biagio Palmisano [3], Emanuela Spica [3], Chiara Tavanti [3], Samuele Di Cristofano [4], Samantha Donsante [5], Marta Serafini [5,6], Tiziana Borsello [7,8], Mary Anna Venneri [1], Alessandro Corsi [3], Debora Salerno [3], Benedetta Donati [9], Alessia Ciarrocchi [9], Domenico Raimondo [† 4], Mara Riminucci [†,* 3]

[1] Department of Experimental Medicine, Sapienza University of Rome, 00161 Rome, Italy

[2] IRCCS Neuromed, 86077 Pozzilli (IS), Italy

[3] Department of Molecular Medicine, Sapienza University of Rome, 00161 Rome, Italy

[4] Department of Molecular Medicine, Sapienza University of Rome, Laboratory Affiliated to Istituto Pasteur-Fondazione Cenci Bolognetti, 00161 Rome, Italy

[5] Tettamanti Center, Fondazione IRCCS San Gerardo dei Tintori, 20900 Monza, Italy

[6] School of Medicine and Surgery, University of Milano Bicocca, 20854 Milan, Italy

[7] Department of Pharmacological and Biomolecular Sciences, University of Milan, 20133 Milan, Italy

[8] Department of Neuroscience, Mario Negri Institute of Pharmacological Research IRCCS, 20156 Milan, Italy

[9] Laboratory of Translational Research, Azienda USL-IRCCS di Reggio Emilia, 42122 Reggio Emilia, Italy

[†] *These authors jointly supervised this work*

* Correspondence to mara.riminucci@uniroma1.it

Keywords: denosumab, bone resorption, bone remodelling, GNAS, Gsα

a

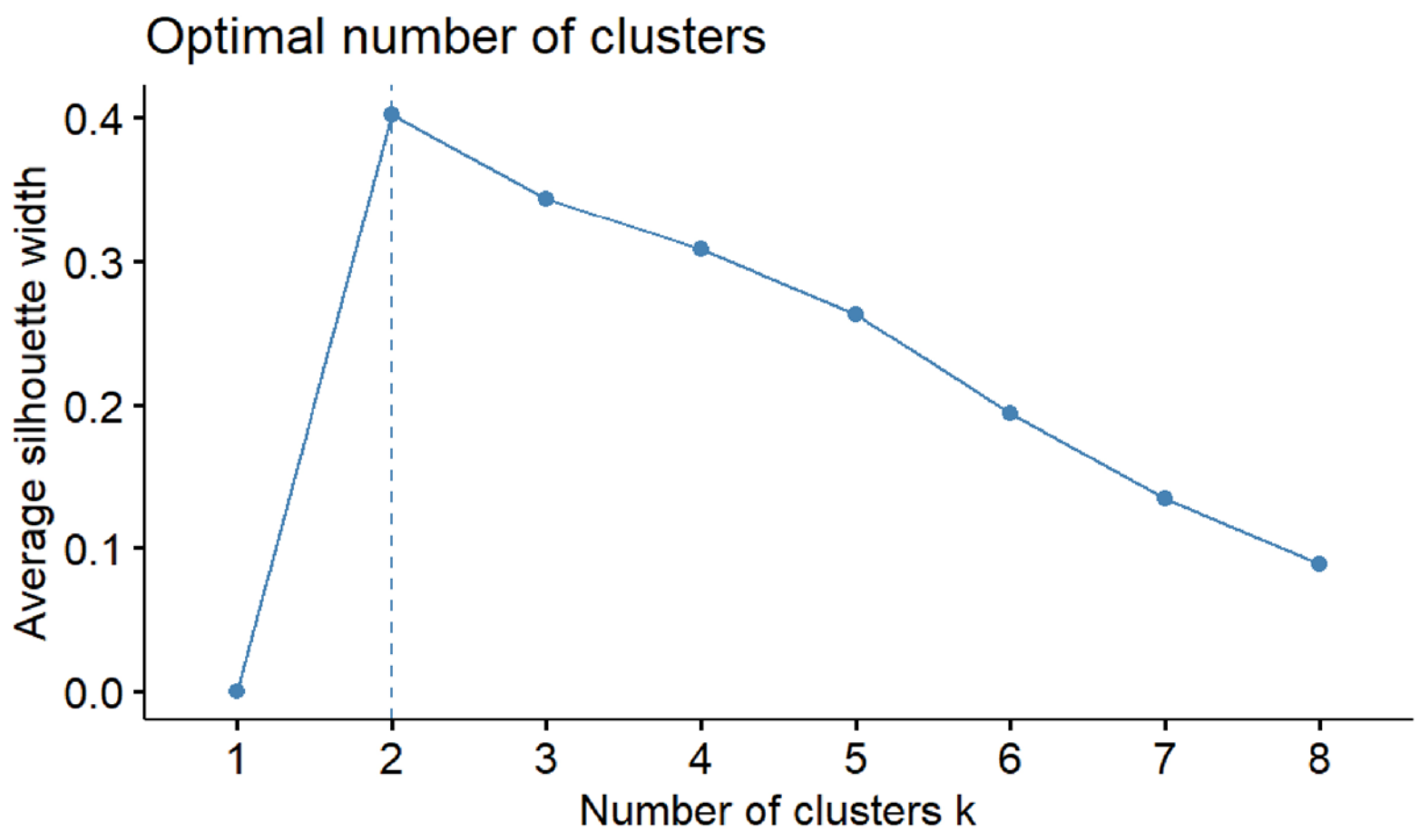

b

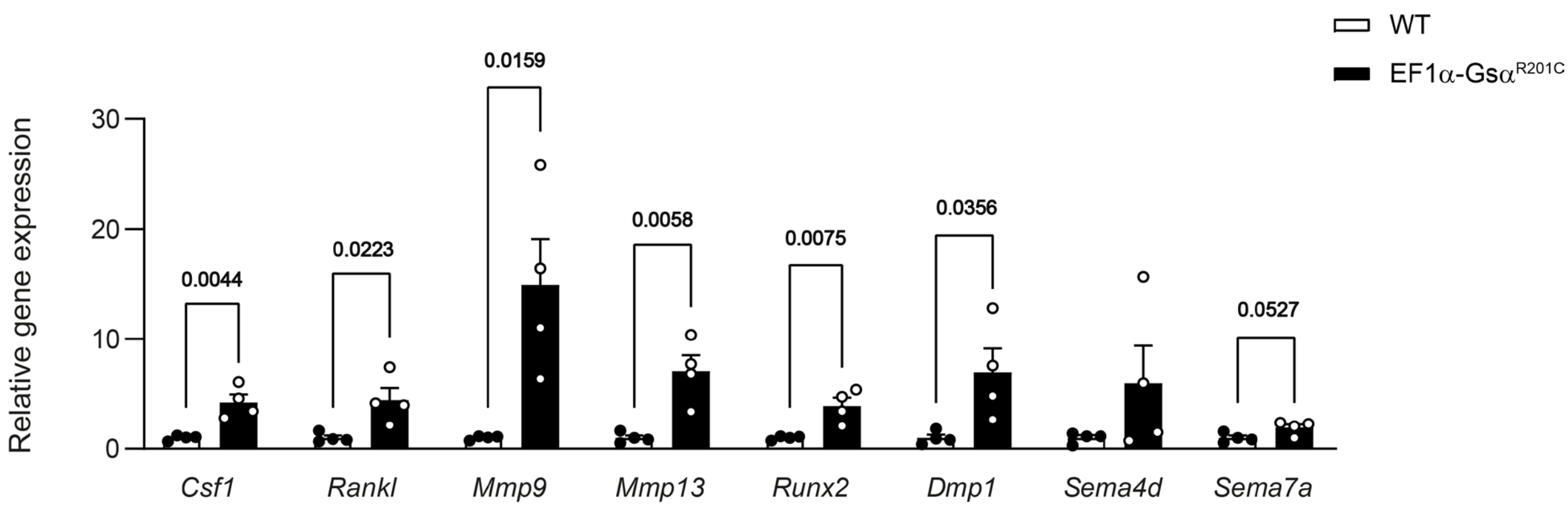

Supplementary Figure 1: Sample segregation and validation of NanoString analysis. a) Silhouette plot analysis showing samples segregation into two clusters. **b**) Gene expression analysis performed on tail vertebrae of 3-month-old WT (n=4 mice) and EF1α-Gsα^{R201C} (n=4 mice) mice. Data are represented as the mean ± SEM. Statistical analysis was performed using Student t-test. The exact P-value is reported on column bars; significance at $P < 0.05$.

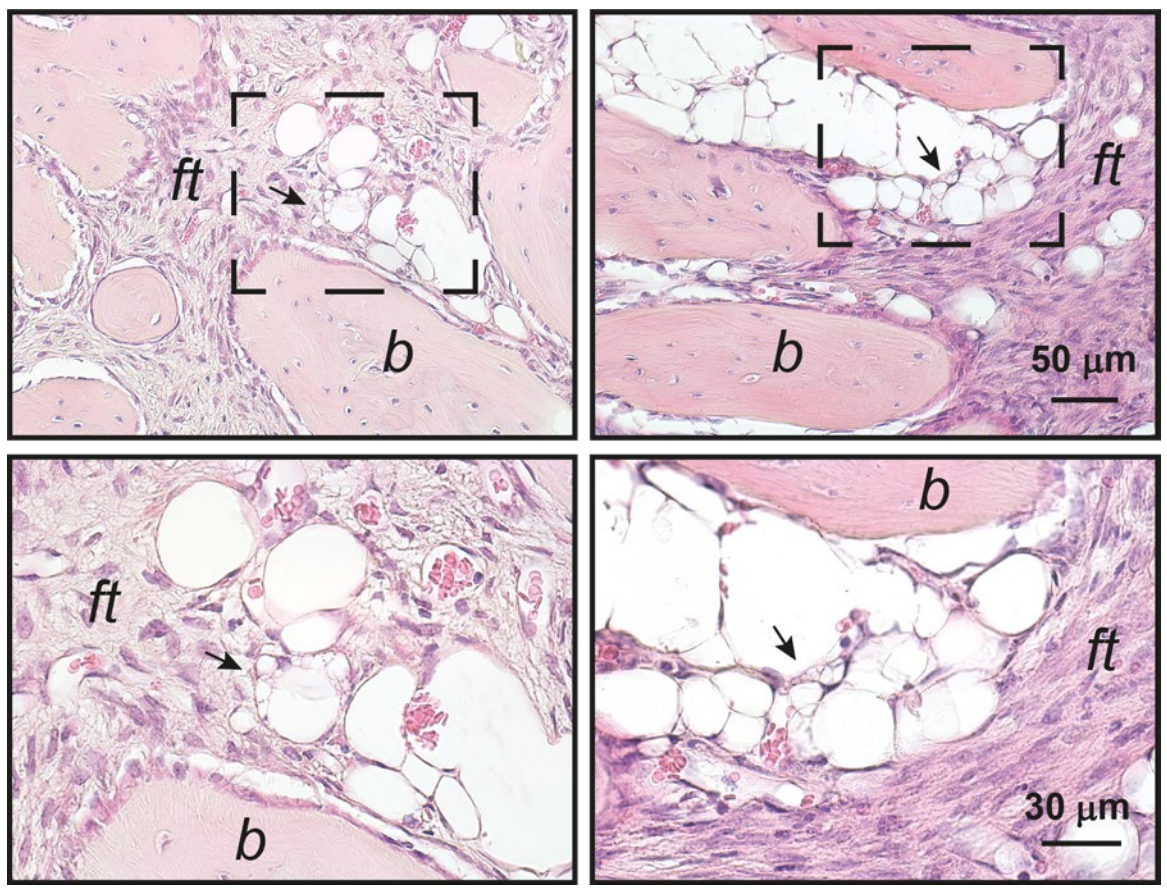

Supplementary Figure 2: Effects of RANKL inhibition on adipocyte differentiation in tail vertebrae of EF1α-Gsα^{R201C} mice. Haematoxylin-Eosin (H&E)-stained tissue sections of tail vertebrae of αRANKL-treated EF1α-Gsα^{R201C} mice showing the development of multilocular adipocytes (arrows). *b* = bone; *ft* = fibrous tissue.

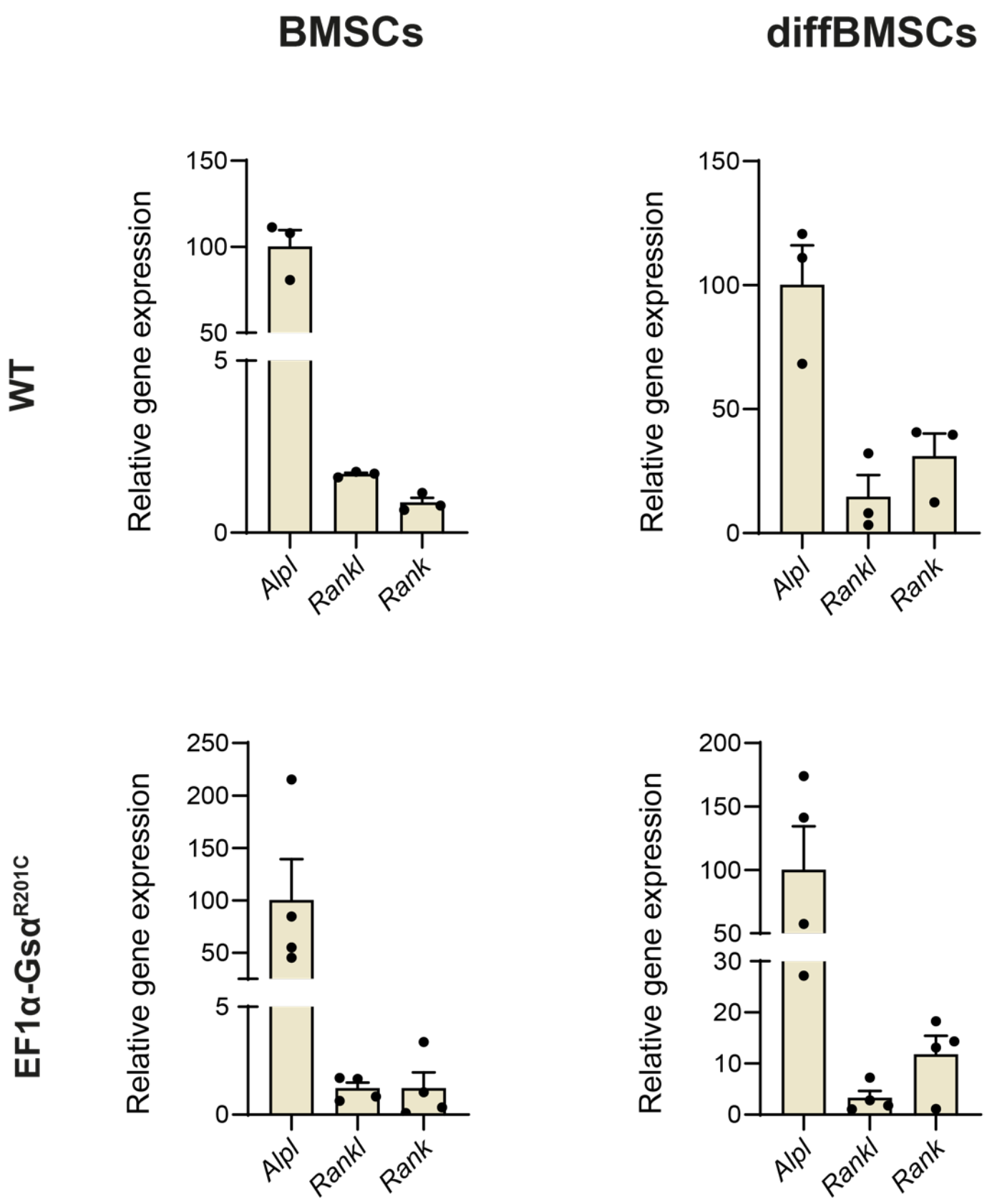

Supplementary Figure 3: Expression of *Rankl* and *Rank* in WT and EF1α-Gsα^{R201C} bone marrow stromal cells (BMSCs). Both transcripts are detected at basal conditions (BMSCs) and after osteogenic induction (diffBMSCs). Their expression level is compared to the osteogenic marker *Alpl*. WT n=3 mice; EF1α-Gsα^{R201C} n=4 mice. Data are represented as the mean ± SEM.
