# Supplementary material for: Exploring bone formation mechanism and pattern during RANKL inhibition in a fibrous dysplasia mouse model
Source: Commun Biol. 2026 Apr 22;9:1088. doi: 10.1038/s42003-026-10097-z (PMC13463084; doi:10.1038/s42003-026-10097-z)
Supplement: Supplementary file 2 [file 42003_2026_10097_MOESM2_ESM.pdf]

Description of Additional Supplementary Files

File name: Supplementary Data 1

Description: Gene expression profiling was conducted using NanoString nCounter platform with a custom CodeSet targeting 54 genes involved in osteoclastogenesis, matrix remodeling, osteoblast-osteoclast cross-talk and osteogenesis

File name: Supplementary Data 2

Description: Numerical source data for graphs and charts are provided as Supplementary Data 2.

File name: Supplementary Data 3

Description: Numerical source data for graphs and charts are provided as Supplementary Data 3.

File name: Supplementary Data 4

Description: Numerical source data for graphs and charts are provided as Supplementary Data 4.

File name: Supplementary Data 5

Description: Numerical source data for graphs and charts are provided as Supplementary Data 5.
